# The Longitudinal Interplay Between Social Network and Psychopathology in Multi-Problem Young Adult Men; Separating Within-and Between-Person Effects

**DOI:** 10.3389/fpsyg.2021.727432

**Published:** 2021-12-09

**Authors:** Loïs Schenk, Miranda Sentse, Reshmi Marhe, Laura van Duin, Godfried Engbersen, Arne Popma, Sabine Severiens

**Affiliations:** ^1^Department of Psychology, Education and Child Studies, Erasmus University Rotterdam, Rotterdam, Netherlands; ^2^Institute of Criminal Law and Criminology, Leiden University, Leiden, Netherlands; ^3^Amsterdam University Medical Center, Amsterdam, Netherlands

**Keywords:** young adulthood, social network, psychopathology, random intercept cross-lagged panel model, multi-problem

## Abstract

Young adulthood is characterized by many life changes. Especially for young men with problems across different life domains (i.e., multi-problem), these changes may entail obstacles. Incidences of psychopathology increase during young adulthood and at the same time important shifts in social networks – such as changing relations with peers and parents, isolation, or deviant peer affiliation – take place. The present study examined the longitudinal interplay between psychopathology and social network characteristics over the course of 1 year in multi-problem young adults, at both between-person and within-person level. A sample of 696 multi-problem young adult men (age 18–27) participated in this three wave study. We used traditional cross-lagged panel models (CLPM) to examine how social network characteristics and psychopathology are related at the between-person level, and random intercept cross-lagged panel models (RI-CLPM) to examine within-person links. Between-person associations between internalizing problems and social networks were bidirectional, and externalizing problems were related to problematic social network characteristics, but not vice versa. At the within-person level, no such cross-lagged paths were found. Overall, results indicated that in multi-problem young adults, social network characteristics and psychopathology are related. However, looking at within-person processes this relation is not reciprocal.

## Introduction

Young adults’ lives are generally marked by the end of education, declines in parental support, and more self-sufficiency. For young adults (18–28 years) the drop in institutional structure and more independence from expected social roles, may result in increased well-being (Schulenberg and Zarrett, 2006). However, at the same time young adulthood is marked by increased incidences of psychopathology which often co-occur with disadvantages and limited resources in multiple domains (Schulenberg and Zarrett, 2006; Bodden and Deković, 2016). Research shows that for so-called multi-problem young adults, problems across different life domains are present, such as delinquent friends, mental health problems, addiction, and personal delinquency (Zijlmans et al., 2021). For these multi-problem young adults, a growing emphasis on self-sufficiency can be overwhelming and may reveal a mismatch between individual needs and contextual resources, which makes the transition to adulthood challenging (Pettit G. S. et al., 2011).

Psychopathology in childhood and adolescence has been studied extensively in its co-occurrence with family- and peer related characteristics of social networks (e.g., Wentzel and Feldman, 1996; Brendgen et al., 2005; Parker et al., 2006; Kochel et al., 2012; Fryers and Brugha, 2013; Maes et al., 2019). During young adulthood, however, social networks may be different from social networks during adolescence. Young adults often move out of the family home, with implications for parent-child relations, friendship networks become smaller but more intensive, and newly formed romantic relationships become part of social networks (Furman et al., 2002; Parker et al., 2006). A healthy social network consists of contacts with people providing emotional encouragement, guidance, and access to information and resources (Thompson and Goodvin, 2016). Having hindering contacts, living in social isolation, and lack of family contact are indications of less healthy, or problematic social networks (Walen and Lachman, 2000; De Jong-Gierveld et al., 2006). These proximal indicators of social networks in young adulthood can offer both protection and risk for psychopathology (Schulenberg et al., 2004). Previous studies, however, have not considered social networks of young adults in relation to both internalizing and externalizing problems. Insight in these relations of multi-problem young adult men is necessary in order to adequately support them.

In the present study, we will address young adult men’s social network as a global concept that includes resources as well as risk factors of social support, both from parents and peers. Using this global concept, we study the interrelation of social networks and psychopathology among multi-problem young adult men. We will also focus on the implications of traditional and advanced statistical methods in studying these relations.

### Explaining the Interrelation Between Psychopathology and Social Networks

There are three models plausible for explaining the interrelation between psychopathology and social networks: (1) the interpersonal risk model, stating that social networks predict psychopathology; (2) the symptoms-driven model in which psychopathology predicts social networks; and (3) the transactional model in which psychopathology and social networks influence each other over time. The three models, including theoretical foundation and empirical evidence (mainly derived from adolescent samples), will be discussed below.

The assumption underlying the *interpersonal risk model* is that psychopathology arises in young adults’ social environment, for example, when youths live in social isolation or have hindering contacts. Socialization is one of the processes explaining this model, and most consistent associations in this regard are explained by the differential association theory (Sutherland, 1947; Matsueda, 2001). This theory states that being part of a group with favorable attitudes toward delinquency provides a context to learn these skills and may encourage this behavior in return. Studies repeatedly show the association between deviant peer affiliations and externalizing problem behaviors through socialization processes (e.g., Brendgen et al., 2000; Haynie, 2002; Brechwald and Prinstein, 2011), also after controlling for selection effects using dynamic social network studies (for a review, see Sijtsema and Lindenberg, 2018). Additionally, socialization of depression seems to arise through processes of co-rumination (for a review, see Spendelow et al., 2017) and failure anticipation (Van Zalk et al., 2010b). Social isolation, that is having a lack of ties or attachment with family and friends (Haynie, 2002) as predictor of psychopathology, is likely to be a significant stressor that undermines the need to belong (Baumeister and Leary, 1995; Saunders et al., 1998). Ultimately, this can lead to or escalate psychopathology in young adulthood (McLewin and Muller, 2006; Holt et al., 2018; Bennett et al., 2019; Matthews et al., 2019). In sum, theoretical basis and empirical evidence of the interpersonal risk model is reflected in studies that associate social network characteristics during childhood and adolescence with subsequent psychopathology.

As opposed to the interpersonal risk model, the *symptoms-driven model* proposes that young adults’ psychopathology precedes the characteristics of their social network (Kochel et al., 2012). The underlying assumption is that individuals with certain characteristics select companionship of similar others. Social control theory (Hirschi, 1969) suggests that adolescents with weak ties to society prefer to associate with friends who are similar to themselves in this. There is a body of research showing that due to self-selection and de-selection, depressive and deviant youths cluster together (Prinstein, 2007; Van Zalk et al., 2010a; Dishion and Patterson, 2015; TenEyck and Barnes, 2015; Franken et al., 2016; Schwartz et al., 2019). Eliciting negative interactions is, next to selection, a way how individuals (unintentionally) shape their own environment. Youths who show depressive symptoms or levels of aggression may elicit negative interactions from their relatives and peers so that relationships cannot be maintained or lead to rejection (Kuwabara et al., 2007; Rudolph et al., 2008; Tseng et al., 2013). To conclude, the relation between psychopathology and subsequent social network characteristics can be explained by the symptoms-driven model. Evidence for this model comes from mechanisms of social deficits, eliciting negative interactions, withdrawal, and self-selection.

Lastly, a combination of the interpersonal risk model and symptoms-driven model is captured by a *transactional model* in which psychopathology and social network characteristics are reciprocally related over time. This model takes interrelations among dynamic systems, such as psychological and sociological systems, into account (Sameroff and Mackenzie, 2003). An implication of this interdependency is that manifestations of psychopathology depend on youths’ social network on the one hand, but that youths’ characteristics partially determine the nature of their network as well. Deviation amplifying processes of both selection and socialization (Sameroff and Mackenzie, 2003), are consistently found in studies on offending behavior among adolescents (for a review, see Gallupe et al., 2019). A growing body of literature considers these reciprocal processes when studying the longitudinal development of psychopathology in childhood and adolescence (see Leve and Cicchetti, 2016). These studies, however, have largely ignored characteristics of social networks during young adulthood. Research that did focus on social network characteristics during young adulthood (proximal characteristics) found mixed results and focused only on peer affiliations (Jones et al., 2016; Samek et al., 2016), or internalizing problems, and was based on a general population sample (Pettit J. W. et al., 2011; Taylor et al., 2014). How proximal indicators of social networks and psychopathology of multi-problem young adults are interrelated over time, remains relatively unclear.

To study psychopathology and social networks in a transactional model framework, both pathways should be studied simultaneously. The aim of the current study, therefore, is to test the direction of effects between young adults’ social networks and psychopathology in the period of 1 year. Since there are different findings on how internalizing and externalizing problems are linked to social networks, we test separately for these two dimensions of psychopathology.

### Simpson’s Paradox

Studying the interrelation between young adults’ social networks and psychopathology will often lead to proposed inferences on the individual level. For example, transactional associations between youths’ antisocial behavior and parental monitoring are translated in suggestions that prevention and intervention programs should not only focus on parenting behaviors, but on youths’ behaviors as well (Wertz et al., 2016). Similarly, Jones et al. (2016) interpret their findings on the link between psychopathology and social environments as support of the importance of intervening in social environments. However, these findings are based on analyses of traditional cross-lagged panel models (CLPM); youths’ rank-order positions, i.e., their scores relative to the group’s mean score, are used to study the relation between two or more constructs. With social networks and psychopathology known to be relatively stable within individuals (Sarason et al., 1986; Ferdinand and Verhulst, 1995), it seems important to consider trait-like individual differences as well. On a within-person level, therefore, youths’ scores relative to their own expected scores can be used to examine interrelatedness of the two constructs. Traditional cross-lagged models do not take the distinction between these two levels (between- and within-person) into account. The strength of associations found in these traditional models is therefore strictly speaking incorrect as a basis for inferences or conclusions at the individual level. Hamaker et al. (2015) proposed a random intercept cross-lagged panel model (RI-CLPM) instead to disentangle the between- and within-person variance in the concepts under study.

Between- and within-person level analyses are suitable for two different types of research questions. Analysis on the between-person level (i.e., the traditional CLPM) will provide insight in the average associations between psychopathology and social networks for a given sample of individuals (e.g., “*Do young adults with more than average problematic social networks also have higher than average levels of psychopathology?”*). When the aim is to identify groups of people who are at risk for psychopathology or problematic social networks, this is a suitable question that can be answered with traditional between-person analysis. Practical implications, however, cannot be drawn from this analysis, since it does not answer the question of how the two constructs are related *within* individuals, where the causal processes actually take place (Keijsers, 2016). To draw accurate inferences for interventions and thus to study mechanisms on the individual (within-person) level, an alternative (RI-CLPM) model is needed that can answer questions such as: “*If young adults, over time, experience an increasing amount of problems in their social network, do their levels of psychopathology then also change accordingly, and vice versa*?”. Previous research has shown that the two questions and associated analytic strategies often result in different outcomes (e.g., Keijsers, 2016; Barzeva et al., 2019). Others even found a reversed association; associations at the between-person level are positive, whereas associations at the within-person level are negative (e.g., Ousey et al., 2011). This phenomenon is referred to as *Simpson’s paradox*, which means that causal inferences drawn from the population level may not be true for subgroups or intra-individual changes (Kievit et al., 2013; Keijsers, 2016). Traditional CLPM models and RI-CLPM models, therefore, should be used appropriately to answer the question at hand.

### The Present Study

The current study will investigate the interrelatedness of psychopathology and social networks in a sample of multi-problem young adult men. Adolescent and young adult males have distinct health risk profiles from females. Moreover, men experience more unmet mental health needs resulting from stigma, cultural expectations, and disengagement with health service (Rice et al., 2018). In order to adequately support these multi-problem young adult men, more knowledge is required on their social network in relation to their psychopathology. To test if and how social networks and psychopathology are related among multi-problem young adults, we will apply traditional cross-lagged panel models (CLPM), separately for internalizing and externalizing problems. In addition, to test if these links are also present at the individual level, and as such may form a starting point for intervention or prevention, we will apply random intercept cross-lagged panel models (RI-CLPM) (Hamaker et al., 2015). The focus on proximal indicators of young adults’ global social networks, and the use of recent methodological advances, make this study exploratory in nature. For each model (between- and within-person level, and for externalizing and internalizing problems) we will test if the results are in line with the *interpersonal risk model, symptoms-driven model*, or *transactional model.*

## Materials and Methods

### Participants and Procedure

The sample comprised 696 multi-problem, ethnically diverse, young adult males between 18 and 27 years old (mean = 22.05, SD = 2.44). Previous research on this sample revealed the high prevalence of (borderline) clinical dysfunction of participants; 42% of the sample reported (borderline) clinical internalizing problems, and 29.9% reported serious externalizing problems (van Duin et al., 2019). Participants were recruited in 2014–2016 at the municipal agency for young adults (18–27) (in Dutch: Jongerenloket) and at a multimodal day treatment program, both in Rotterdam, The Netherlands (for details on the recruitment and the multimodal day treatment program, see (Luijks et al. (2017)). The first site of recruitment, the municipal agency, is where young adults can apply for social welfare and, if needed, can be referred to a treatment program. Young adults at this site were eligible for participation in the study if they were male, aged between 18 and 27 years, and met the criteria of a multi-problem young adult. Participants’ multi-problem status was assessed by the Self-Sufficiency Matrix – Dutch version (Fassaert et al., 2014). Eleven life domains are scored ranging from 1 “acute problems” to 5 “completely self-sufficient.” Participants were considered multi-problem when they met the following criteria: (a) a score of 1 or 2 on the domains Income and Daily activities, (b) a score of 1, 2, or 3 on at least one of the following domains: Addiction, Mental health, Social network, Justice and (c) a score of 3, 4, or 5 on the domain Physical health (Luijks et al., 2017). The 7 domains used to consider participants as multi-problem in the first recruitment site were selected based on their match with the domains that are explicitly targeted by the intervention program in the second recruitment site. As such, the two subpopulations were considered multi-problem based on the same definition. Domains such as “Domestic relations,” “Community involvement,” and “General life skills” were not considered to be part of the definition of multi-problem. Cut-off scores were established based on the lack of self-sufficiency in all domains (i.e., a maximum score of 2 or 3), but for pragmatic reasons, participants had to be self-sufficient regarding physical health (i.e., a minimum score of 3).

The second site of recruitment, the multimodal day program *New Opportunities* (in Dutch: De Nieuwe Kans, DNK), is a program specifically developed for multi-problem young adult males. Participants could have been referred to the program by the municipal agency for young adults, youth care, probation services, or social organizations, or could have entered the program on their own initiative. Young adults recruited at this site were in any case eligible for participation, since the program was aimed at the same target population as the research. 177 participants were recruited at this second site and 519 participants were recruited at the municipal agency. The recruitment places did not indicate any specific treatment; most of the respondents were in some sort of treatment program (about 20 different ones) but these programs were not explicitly aimed at either social networks or psychopathology. As such, all 696 respondents were part of our one sample of multi-problem young adult men.

After providing oral and written information by one of the researchers, individuals could decide if they wanted to participate. When they did, participants gave written informed consent. Trained researchers provided participants with questionnaires which they orally assessed or, in the case of sensitive topics such as delinquency or childhood trauma, participants were offered to fill out those questionnaires themselves. The confidentiality of the respondents was maintained throughout the study. There were four waves in which interviews were conducted with the participants. For the first wave, interviews were conducted within the first 4 weeks after intake at the municipal agency or in the first 2 weeks after start of the day treatment program. The second, third and fourth wave were conducted 2–4 months after baseline, 6–8 months after baseline, and 12–14 months after baseline, respectively. These interviews were conducted at the municipal agency, the day program, the research site, juvenile justice facility or detention center, at a participant’s home, or at a public space. Consistency in interview contexts between researchers and waves was taken into account by extensive training and observation. Of the 696 participants at the first wave, 73% (*n* = 513) participated in the second wave, 70% (*n* = 485) in the third wave, and 78% (*n* = 542) in the fourth wave. Based on our selection of measures, the present study will only use data of the first (here: T1), third (here: T2), and fourth (here: T3) wave. 64% of all participants in the first wave was present at all three waves, 19% was present at two waves, and 17% was present at only one wave. On average, participants with missing values on T2 did not significantly differ in their social network scores at T1 from respondents to T2 [difference −0.03, 95% CI (−0.15 −0.08), *t*(1,063) = −0.57, *p* = 0.570]. Neither did they differ from T2 respondents in externalizing problems scores on T1 [difference −2.52, 95% CI (−5.76 −0.71), *t*(1,067) = −1.53, *p* = 0.126]. Participants with missing values on T2 did differ from T2 respondents in their internalizing problem scores on T1. They had lower internalizing problem scores (*M* = 65.40, SE = 1.38) than respondents on T2 (*M* = 69.16, SE = 1.03). This difference of −3.76 was significant [95% CI (−7.14 −0.39), *t*(857) = −2.19, *p* = 0.03]. This would indicate a possible underestimation of internalizing problems in our sample. Participants who did not fill in T3 did not differ on any of the studied T1 variables compared to respondents to T3 {T1 Social network scores [difference −0.08, 95% CI (−0.20 −0.05), *t*(1,063) = −1.18, *p* = 0.238], T1 internalizing problem scores [difference −0.08 95% CI (−5.87 –1.25), *t*(1,067) = −1.27, *p* = 0.203], T1 externalizing problem scores [difference 0.02, 95% CI (−3.46 –3.50), *t*(1,067) = 0.01, *p* = 0.990]}. We included all 696 participants in our study irrespective of whether they provided data at all these three waves (see section “Analyses”).

The design of this study has been approved by the Medical Ethical Review Committee of the VU University Medical Center (registration number: 2013.422 – NL46906.029.13).

### Measures

#### Psychopathology

Psychopathology was assessed by the Adult Self Report (ASR; Achenbach and Rescorla, 2003). The ASR comprises 123 items rated on a 3-point scale ranging from *not true* to *always true*, measuring psychological health outcomes and social adaption. Internalizing and externalizing problems are two distinguished dimensions. The internalizing scale consist of three subscales: somatic complaints, anxious/depressed, and withdrawal. The externalizing scale consists of the subscales: intrusive, rule-breaking and aggressive behavior. Total problem scores for both scales are calculated by adding up individual item scores. These broadband scales internalizing and externalizing problems were used as outcomes measures. The internal consistency of the questionnaire is good, with a Cronbach’s α of 0.85 for internalizing problems and α of 0.88 for externalizing problems. The ASR was administered at T1, T2, and T3.

#### Social Network

Social network was assessed by the Dutch version of the Self sufficiency matrix (SSM-D), filled out by the researcher (Fassaert et al., 2014). The SSM is often used as an assessment tool of acceptable functioning in several domains, expressed in levels of self-sufficiency (Bannink et al., 2015). In terms of convergent validity, the SSM-D correlates significantly and positively with two measures of mental and social health: HoNOS; Health of the Nations Outcomes Scale and CANSAS; Camberwell Assessment of Needs Short Appraisal Schedule (Fassaert et al., 2014). Social support in the social network domain was measured by a single item, rated on a five point scale: 1 = “acute problem,” 2 = “not self-sufficient,” 3 = “barely self-sufficient,” 4 = “adequately self-sufficient,” and 5 = “completely self-sufficient.” *Lack of necessary support from family / friends and no contacts other than possibly deviant friends or serious social isolation*, was rated as “problematic,” whereas “completely self-sufficient” implied a healthy social network. See Appendix A for the full scale. The SSM was completed by researchers at the end of the test-battery so that the information of the prior questionnaires could be used to validly assess the SSM. The SSM was administered at all four waves, of which we used T1, T2, and T3.

### Analyses

Our research questions were examined through cross-lagged path modeling in Mplus 7.4 (Muthén and Muthén, 2012). First, we applied traditional cross-lagged panel models. Second, to differentiate between- and within-person level relations, we used random intercept cross-lagged panel models, as suggested by Hamaker et al. (2015). For the latter we created within-person centered variables per construct per wave and random intercepts for psychopathology and social networks, to take stable differences into account (Hamaker et al., 2015). For both analytic strategies we conducted analyses separately for internalizing and externalizing problems. We kept both models as equal as possible in terms of constraining, which allowed us to compare the models and to attribute possible differences to the random intercepts. As a sensitivity check, we used Satorra-Bentler difference tests to see whether model constraints (in groups of paths) would lead to significant differences in model fit. Fully constrained models were favored in terms of model parsimony. This fully constrained model had the following constraints: autoregressive stabilities were fixed to be the same across the three waves for psychopathology, and also for social networks; the cross-lagged paths from psychopathology to social networks were fixed to be the same across the waves, and the cross-paths from social network to psychopathology too. Within-time co-variances between psychopathology and social networks were constrained to be equal for all three waves. We evaluated final model fit on the basis of Root Mean Square Error of Approximation (RMSEA), comparative fit index (CFI), and Standardized Root Mean Square Residual (SRMR) (Little, 2013). CFI higher than 0.95, RMSEA below 0.06, and SRMR below 0.08 are indicative of a good model fit (Hu and Bentler, 1999). We estimated the stability in our variables, the within-time correlations, the bidirectional (cross-lagged) paths, and the intercepts of psychopathology and social networks.

To handle missing data and to account for somewhat skewed distributions of psychopathology, we used the Maximum Likelihood with Robust errors (MLR) estimation, in so that all available observations of the total sample are being used.

To split the variance of constructs into between-person and within-person, sufficient variance at the within-level is necessary. Therefore, we calculated intra-class correlations (ICC) of each variable. ICC of internalizing problems was 0.56, which indicates that 56% of the variance in the three measurement points is explained by between-person differences, and the remaining 44% by within-person fluctuations. ICC of externalizing problems was 0.65, and for social network 0.26. A substantial part of the variance in internalizing and externalizing problems is due to stable between-person differences, but still 44% and 35%, respectively, is due to individual fluctuations over time. For social network this is even 74%. This is sufficient reason to apply both the traditional and random intercept models. We compared the fit between the two types of models with the Satorra-Bentler scaled χ^2^ difference test.

## Results

### Descriptive Statistics

Table 1 shows the means and standard deviations of all study variables, for all three waves. Social network scores in general increased, but the healthiest reported social network was at T2. Internalizing problems were higher than externalizing problems at all time points. Both internalizing and externalizing problems decreased over time.

**TABLE 1:** Means and standard deviations of social network and psychopathology.

	*Mean*	*SD*	*Min-Max*	*n*
Social network T1	3.35	0.95	1–5	690
Social network T2	3.62	1.00	1–5	475
Social network T3	3.57	0.91	1–5	447
Internalizing problems T1	68.88	26.55	2–99	692
Internalizing problems T2	61.33	29.67	2–99	477
Internalizing problems T3	60.12	29.85	2–99	534
Externalizing problems T1	65.08	25.86	2–99	692
Externalizing problems T2	59.18	27.79	2–99	477
Externalizing problems T3	57.43	28.50	2–99	534

Bivariate correlations among the variables are displayed in Table 2. Internalizing and externalizing problems were each significantly and negatively correlated with social network at all three time points: lower social network scores were associated with higher internalizing and externalizing problems. Internalizing and externalizing problems were significantly positively correlated with each other at all three time points.

**TABLE 2:** Correlations between social network and psychopathology.

	1	2	3	4	5	6	7	8	9
1. Social network T1									
2. Social network T2	0.33**								
3. Social network T3	0.32**	0.32**							
4. Internalizing problems T1	−0.31**	−0.28**	−0.22**						
5. Externalizing problems T1	−0.23**	−0.26**	−0.20**	0.64**					
6. Internalizing problems T2	−0.26**	−0.36**	−0.27**	0.67**	0.48**				
7. Externalizing problems T2	−0.17**	−0.33**	−0.19**	0.50**	0.72**	0.69**			
8. Internalizing problems T3	−0.25**	−0.30**	−0.34**	0.62**	0.42**	0.75**	0.54**		
9. Externalizing problems T3	−0.16**	−0.23**	−0.27**	0.44**	0.67**	0.52**	0.76**	0.65**	

Spearman’s rho **p < 0.01.

### Cross-Lagged Path Models

To be able to compare the traditional and RI-CLPMs, we kept model constraints equal between models. Models were run and compared for internalizing and externalizing problems separately. As a sensitivity check, we tested whether freeing the paths across and within the time points would increase model fit, but this was not the case. For internalizing problems, freeing the cross-lagged, stability, or concurrent paths did not lead to a significantly better model fit in the traditional models [respectively, χ^2^ (2) = 1.57, *p* < 0.01; χ^2^ (2) = 0.63, *p* < 0.01; χ^2^ (1) = 1.57, *p* < 0.01], and neither in the RI-CLPM [χ^2^ (2) = 2.00, *p* < 0.01; χ^2^ (2) = 2.48, *p* < 0.01; χ^2^ (1) = 1.67, *p* < 0.01, respectively]. For externalizing problems, freeing the cross-lagged, stability, or concurrent paths did not lead to better model fit either in the traditional models [respectively, χ^2^ (2) = 1.66, *p* < 0.01, χ^2^ (2) = 0.53, *p* < 0.01, χ^2^ (1) = 1.44, *p* < 0.01], or in the RI-CLPMs [χ^2^ (2) = 1.06, *p* < 0.01, χ^2^ (2) = 4.49, *p* < 0.01, χ^2^ (1) = 2.27, *p* < 0.01]. Our fully constrained traditional models showed acceptable model fit, for both internalizing and externalizing problems (see Table 3). Our fully constrained RI-CLPM showed good model fit for both internalizing problems and externalizing problems. Satorra-Bentler comparisons of the traditional and RI-CLPM models revealed significant differences in model fit. The model including random intercepts fitted the data significantly better than the traditional model, for internalizing problems [χ^2^ (3) = 31.22, *p* < 0.001] and for externalizing problems [χ^2^ (3) = 46.86, *p* < 0.001].

**TABLE 3:** Fit indices for traditional and random intercept cross-lagged path models.

Model		RMSEA (90% CI)	CFI	SRMR
**Internalizing problems**				
	Traditional CLPM	0.071 (0.050–0.094)	0.955	0.045
	RI-CLPM	0.000 (0.000–0.049)	1.000	0.025
**Externalizing problems**				
	Traditional CLPM	0.088 (0.067–0.110)	0.94	0.052
	RI-CLPM	0.009 (0.000–0.051)	1.000	0.029

*RMSEA, Root Mean Square Error of Approximation; CFI, Comparative Fit Index; SRMR, Standardized Root Mean Square Residual.*

### Internalizing Problems

#### Traditional Cross-Lagged Panel Models

**FIGURE 1:**
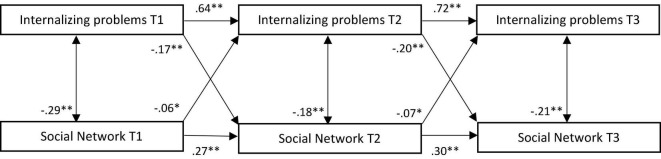
Standardized associations between internalizing problems and social network in traditional model. T, time; ^**^p < 0.001; *p < 0.01.

All stability paths were significant and positive, and concurrent associations between social network and internalizing problems were significant and negative, indicating that better social network scores were related to lower levels of internalizing problems (see Figure 1). Small negative cross-lagged effects were found in both directions between internalizing problems and social network; higher levels of internalizing problems at T1 and T2 were predictive of more problematic social networks at T2 and T3, and more problematic social networks at T1 and T2 were predictive of more internalizing problems at T2 and T3.

#### Random Intercept Cross-Lagged Panel Models

**FIGURE 2:**
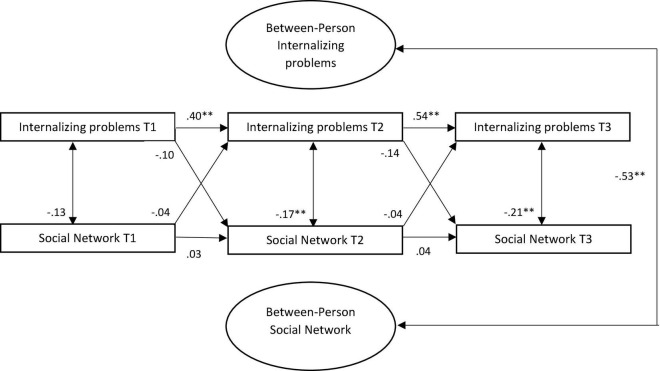
Standardized associations between internalizing problems and social network in RI-CLPM. T, time; **p < 0.001.

At the between-person level, there was a strong negative correlation between stable-traits of social networks and internalizing problems (β = −0.53, *p* < 0.001) (see Figure 2). Young adults with more problematic social networks across the three waves also reported higher levels of internalizing problems across the three waves. At the within-person level, stability paths were only significant for internalizing problems. That is, young adults’ individual deviation in the level of internalizing problems is predicted by their prior deviation from their internalizing problems scores. The social network stability paths were not significant, indicating intra-individual changes over time. There were no significant concurrent associations between one’s social network and internalizing problems at T1. Concurrent associations at T2 and T3, however, suggested that young adults reported higher levels of internalizing problems when their social network scores were low. Moreover, there were no cross-lagged effects between internalizing problems and social network. That is, within-person change in internalizing problems was not predicted by scores on social networks assessed 6 months earlier, and vice versa.

### Externalizing Problems

#### Traditional Cross-Lagged Panel Models

**FIGURE 3:**
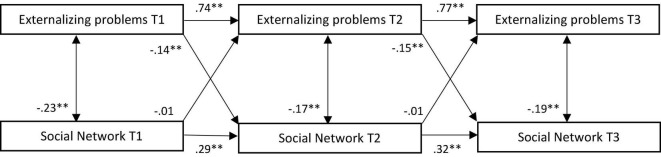
Standardized associations between externalizing problems and social network in traditional model. T, time; ^**^p < 0.001.

All stability paths were significant and positive, and concurrent associations between social network and externalizing problems were significant and negative (see Figure 3). Negative cross-lagged effects were present from externalizing problems to social network, but social network did not predict externalizing problems over time. Higher levels of externalizing problems, thus, were predictive of more problematic social networks 6 months later but not vice versa. These effects, however, were relatively small.

#### Random Intercept Cross-Lagged Panel Models

**FIGURE 4:**
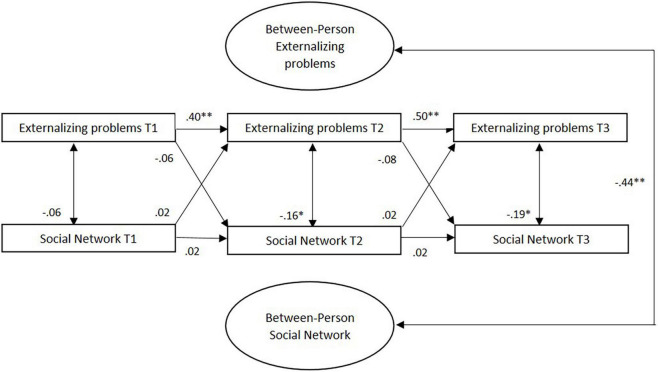
Standardized associations between externalizing problems and social network in RI-CLPM. T, time; ^**^p < 0.001; *p < 0.01.

Correlations between the random intercepts, the between-person effects, were significant. That is, young adults with healthier social networks across the three waves reported lower levels of externalizing problems (β = −0.44, *p* < 0.001) (see Figure 4). At the within-person level, stability paths were only significant for externalizing problems, not for social networks; Young adults’ individual deviation in the level of externalizing problems is predicted by their prior deviation from their own (expected) externalizing problems scores. Concurrent associations at T2 and T3 suggested that young adults reported higher levels of externalizing problems when their social network score was low. There were, however, no significant concurrent associations at the within-person level between social network and externalizing problems at T1. Moreover, in accordance with our findings on internalizing problems, there were no cross-lagged effects between externalizing problems and social network. In other words, within-person change in externalizing problems was not predicted by scores on social networks assessed 6 months earlier, and vice versa.

## Discussion

Given the importance of the transition to adulthood, the current study investigated the interrelatedness of psychopathology and (un)healthy social networks among multi-problem young adult men. We tested three theoretical perspectives on this interrelation over time, using two analytic strategies. First, we applied traditional cross-lagged path models (CLPM) to assess the interrelatedness of social network and psychopathology at a group (between-person) level. Second, we applied random intercept cross-lagged path models (RI-CLPM’s) to disentangle within-person associations from between-person associations. Overall, our results indicated that for multi-problem young adults, social networks and psychopathology are related, but there is no reciprocal relation within individuals.

To study the relation between psychopathology and social networks in multi-problem young adults, we first applied traditional CLPM. In line with previous research, psychopathology and social networks appeared to be stable over time (Sarason et al., 1986; Ferdinand and Verhulst, 1995), and were negatively associated at all three time points. For internalizing problems, we found evidence for the *transactional model* where internalizing problems and social networks were reciprocally related over time; young adults with higher levels of internalizing problems, had more problematic social networks 6 months later than those with lower levels of internalizing problems. Vice versa, young adults with more problematic social networks reported higher levels of internalizing problems over time than those with more healthy social networks. For externalizing problems, we found evidence for the *symptoms-driven model* in which externalizing problems preceded (un)healthy social networks, but social networks did not predict externalizing problems 6 months later.

Using RI-CLPM, we took stable between-person differences into account when testing for within-person processes. Psychopathology was stable over time, but social networks were not. Most importantly, there were no cross-lagged effects at the within-person level, indicating that social networks and psychopathology were related, but change in one construct at the individual level, did not predict change in the other construct. Looking at the within-person level, thus, no evidence was found for any of the three suggested theoretical perspectives regarding the link between psychopathology and social network. In other words, even though there is a co-occurrence of psychopathology and social network, there is no reciprocal relation between the two constructs within persons.

Our results from the traditional models are consistent with previous studies, showing the longitudinal link between social environments and psychopathology (see Leve and Cicchetti, 2016). Previous studies mainly focused on younger age groups, predicting psychopathology and social networks from childhood to adolescence or young adulthood. The present study adds for multi-problem young adults, taking proximal indicators of their social networks into account, that these relations appear to be true as well. However, these relations could not be explained by the transactional and symptoms-driven model. That is, when looking at within-person changes in psychopathology and social network, our study showed that individual processes did not occur as the proposed theories suggested.

Finding different associations on population level and individual level, referred to as Simpson’s paradox (Kievit et al., 2013), has been demonstrated several times in studies that analyzed social environments (e.g., parenting and social support) and individual characteristics (e.g., posttraumatic stress and social anxiety) (see i.e., Birkeland et al., 2016; Keijsers, 2016; Mastrotheodoros et al., 2019; Nelemans et al., 2019). These findings hint on the more complex nature of psychopathology and social networks in itself, and their association. The longitudinal association between social networks and psychopathology may be best explained by stable individual differences in psychopathology, rather than reciprocal processes within persons. One explanation of this finding may be that individual change in social networks or psychopathology needs more time to develop. In our study, the two time lags comprised 6 months each, and 1 year in total. We did additional analyses to see if T1 constructs predicted T3 constructs, but this was not the case. It may be that the within-person effect of change in social network on psychopathology, and vice versa, takes longer than 1 year. Another explanation may be that omitted variables explain both constructs. For example, parental psychopathology influences both children’s psychopathology (biological/genetic; direct/indirect) (Gregory and Eley, 2007; Faro et al., 2019) and the level of positive affect young adult men hold toward their parents (Walker and McKinney, 2015). Additionally, multi-problem young adults are characterized by high levels of adverse childhood experiences (ACEs), and ACEs such as emotional abuse, may also explain both young adults’ psychopathology and relations with their parents (van Duin et al., 2019). Psychopathology and social networks then do not influence each other, but they may be both explained by parents’ psychopathology or ACEs. Also, maybe early maladaptation has led to both psychopathology and problematic social networks during young adulthood, a case of multifinality (Schulenberg and Zarrett, 2006). In this case not proximal indicators of social networks are predictive of psychopathology, but early life predictors are leading to various expressions of adaptational outcomes during young adulthood.

The stability of one’s psychopathology compared to the group tends to be moderate to high between adolescence and adulthood (Schulenberg and Zarrett, 2006). In our study, young adults’ psychopathology was moderately stable over time and stability at the individual level was small to moderate. Noteworthy is the non-stability of young adults’ social networks on the individual level; social network was not related to social network 6 months later. This is supported by the calculated intraclass correlation, which indicated a large part of the variance in social networks lays within individuals, rather than between persons. Consistent with the numerous changes during young adulthood, our results indicate that multi-problem young adults’ social networks are subject to change during this time.

### Strengths and Limitations

The main strengths of the present study include the high number of participants and the longitudinal design which enabled us to study both bidirectional paths simultaneously. The use of proximal indicators of social networks of this developmental stage provided new insights in the link between psychopathology and social network in this specific sample. We used recent methodological insights to separate within- from between-person processes.

However, when reviewing the results of this study some limitations must be kept in mind. First, we used a global conceptualization of social networks. The presence of family support and the presence of deviant peers were taken together in one score, and as such could not be separated. Family support and deviant peer affiliations may be differentially linked to psychopathology (Ioannou et al., 2019). It is therefore also hard to interpret the non-stability in social networks. In addition, social networks are not only defined by the presence or absence of contact, but also by the quality of these contacts. In future research a more comprehensive measure of social support should also include an index of perceived social support. Acceptance, empathy, and support are of great importance during young adulthood (Aquilino, 2006), and this goes beyond how we measured social networks. Furthermore, the sample in this study comprised young adults from diverse ethnical backgrounds and diverse risk profiles (Zijlmans et al., 2021). Future research should evaluate these factors as cofounders of the associations under study. For example, research shows that parent-child differences in acculturation lead to more parent-child stress and conflict, and that this, in turn, is related to ethnic minority adolescents’ psychopathology (Gonzales et al., 2018). Therefore, differences in ethnic diverse backgrounds and the role of cultural gaps between parents and children need to be considered in follow-up research. As the present study was aimed at multi-problem young adult men, future research should include multi-problem women. Relations between women’s social networks and psychopathology, may be different compared to men. For example, women report more social support than men, and women have more favorable attitudes to help seeking compared to men (Dalgard et al., 2006; Yousaf et al., 2015). This may buffer the relation between social networks and psychopathology for women. Future research should include multi-problem women to validate these assumptions. Finally, to establish causal relations, designs studying longer periods of time (i.e., more than 1 year) and using randomized intervention studies, may offer new insights into how psychopathology and social networks are related. It may very well be that these constructs need more time to develop, especially with increasing age. Findings based on randomized controlled trials showing that social networks can improve by using psychosocial interventions, are encouraging (Anderson et al., 2015). It, however, remains the question if targeting psychopathology alone, will impact the quality of social networks.

### Practical Implications

Regarding practical implications, our study showed the importance of taking into account the co-existence of problematic social networks and psychopathology in multi-problem young adults. In most interventions treating psychopathology in young adults, not much attention is given specifically to social networks. To address social support of parents, peers and partners during young adulthood, Functional Family Therapy (FFT) and multidimensional family therapy (MDFT) could be of great importance. Both have been found effective in reducing internalizing and externalizing psychopathology during adolescence by taking social relations in multiple systems into account (Alexander et al., 2013; Van der Pol et al., 2017). Assessing interaction patterns, family structure, and underlying values in relevant systems should be incorporated in the treatment of psychopathology in young adults, but to date, studies of these approaches in young adults are scarce (see Livesey and Rostain, 2017).

Young adults’ mean scores on social networks were low, indicating that many did not have networks which were considered healthy (i.e., supportive contacts and no hindering contacts). In becoming self-sufficient, social networks play a considerable part and need attention. The decline of institutional structure during young adulthood may demand more attention for young adults experiencing either problematic social networks, or psychopathology. Mentors may provide these young adults with additional support. For older youths aging out of care, the presence of a non-parental adult (e.g., a mentor) is believed to improve psychosocial and behavioral outcomes and may as well serve multi-problem young adults (Thompson et al., 2016). The interrelation of problems at multiple life domains is becoming more evident, but still needs more research to support these young adults effectively. Traditional models show the co-occurrence of multiple problems, therefore integrated interventions that address multiple domains are necessary (Osgood et al., 2010; Bannink et al., 2015). However, as our research partly illustrated, individual mechanisms of the emergence and development of multi-issues should not be neglected in interventions.

## Conclusion

The current study provided insight in the relation between proximal indicators of young adult men’s social networks, and their psychopathology within the time span of 1 year. Psychopathology and social network characteristics such as contact with family, isolation, and deviant friends, are interrelated in multi-problem young adult men. Associations between psychopathology and problematic social networks, however, are mostly due to stable between-person differences. Although the patterns shown in the current study support the idea that more problematic social networks elicit psychopathology, and vice versa, this association cannot be explained by individual processes. Therefore, future research should study wider time frames and include third variables that may explain the co-existence of unhealthy social networks and psychopathology within persons. Whereas young adulthood offers increasing opportunities for a person-context match for most young adults (Schulenberg and Zarrett, 2006), limited resources and opportunities are present for multi-problem young adult men. Problematic social networks may not be the cause of psychopathology, but healthy social networks do play a role in healthy development and a successful transition to adulthood. Emotional and material support are needed to enhance the young adult’s life chances (Aquilino, 2006). These insights require a review of present policies and interventions that emphasize the role of social networks and self-sufficiency in general of young adults.

## Data Availability Statement

The raw data supporting the conclusions of this article will be made available by the authors, without undue reservation, to any qualified researcher. Requests to access these datasets should be directed to RM, Marhe@essb.eur.nl.

## Ethics Statement

The studies involving human participants were reviewed and approved by Medical Ethical Review Committee of the VU University Medical Center (registration number: 2013.422 – NL46906.029.13). The patients/participants provided their written informed consent to participate in this study.

## Author Contributions

RM, LD, and AP contributed to conception and design of the study. RM organized the database. LS and MS performed the statistical analysis. LS wrote the first draft of the manuscript under supervision of MS. GE and SS wrote sections of the manuscript. All authors contributed to the manuscript, read, and approved the submitted version.

## Conflict of Interest

The authors declare that the research was conducted in the absence of any commercial or financial relationships that could be construed as a potential conflict of interest.

## Publisher’s Note

All claims expressed in this article are solely those of the authors and do not necessarily represent those of their affiliated organizations, or those of the publisher, the editors and the reviewers. Any product that may be evaluated in this article, or claim that may be made by its manufacturer, is not guaranteed or endorsed by the publisher.

## Appendix

**APPENDIX A:** Social network domain self-sufficiency matrix (SSM-D; Fassaert et al., 2014).

Social network domain	Acute problems	Not self-sufficient	Barely self-sufficient	Adequately self-sufficient	Completely self-sufficient
	Serious social isolation	Few family contact	Some family contact	Sufficient contact with family	Healthy social network
	Lack of family contact	Barely no supportive contacts	Some supportive contacts	Sufficient supportive contacts	Many supportive contacts
	Lack of necessary supportive contacts and no contacts other than possible bad friends	Many hindering contacts	Few hindering contacts	Barely no hindering contacts	No hindering contacts
